# Knowledge, attitude, and practice of nurses in ICU on preventing ventilator-associated pneumonia: a cross-sectional study in Gansu Province, China

**DOI:** 10.3389/fmed.2025.1591582

**Published:** 2025-07-09

**Authors:** Xiarong Li, Xiaoliang Zhang, Yujuan Feng, Ziyan Yao, Yi Sun, Lingna Zhang, Haojun Zhang, Junling Wang

**Affiliations:** ^1^School of Public Health, Lanzhou University, Lanzhou, Gansu, China; ^2^Department of Public Health and Hospital Infection Management, Hospital of Northwest Minzu University (Second People’s Hospital of Gansu Province), Lanzhou, Gansu, China; ^3^School of Public Health, Gansu University of Chinese Medicine, Lanzhou, Gansu, China

**Keywords:** ventilator-associated pneumonia, ICU, knowledge, attitude, practice

## Abstract

**Introduction:**

Ventilator-associated pneumonia (VAP) is a common and life-threatening complication in ICU patients, with its occurrence closely related to ICU nurses’ knowledge, attitudes, and practices. This study aimed to investigate the current status and influencing factors of ICU nurses’ knowledge, attitudes, and practices regarding VAP prevention in Gansu Province, and to provide a basis for improving the effectiveness of VAP prevention.

**Methods:**

A stratified random sampling method was used to select 600 ICU nurses from 24 hospitals in Gansu Province as study participants. Data related to VAP prevention among the nurses were collected through a questionnaire survey and statistically analyzed using SPSS 26.0.

**Results:**

The overall score of nurses on VAP prevention was 113.92 ± 8.472, with the lowest score in the knowledge dimension (7.66 ± 1.200) and higher scores in the attitude (28.67 ± 3.528) and practice (77.59 ± 5.839) dimensions. Factors such as region, ICU type, number of training sessions, and years of work experience significantly affected VAP prevention knowledge, attitudes, and practices. The knowledge scores of nurses in the Lanzhou and Zhangye regions were significantly higher than those in other regions, and nurses in comprehensive ICUs had higher VAP knowledge scores. Additionally, nurses who participated in four or more VAP prevention training sessions had significantly higher scores in all dimensions compared to those with fewer training sessions. A significant positive correlation was found between knowledge, attitudes, and practices related to VAP prevention.

**Conclusion:**

ICU nurses in Gansu Province performed better in the attitudes and practices of VAP prevention, but there is still room for improvement in their knowledge. Regional differences, ICU type, and the number of training sessions are important factors influencing VAP prevention and control abilities. The study suggests that enhanced training in VAP prevention and control knowledge can help improve nurses’ attitudes and practical skills. To improve the effectiveness of VAP prevention, hospitals should focus on strengthening training in areas with weaker knowledge and increasing the frequency of training sessions.

## 1 Introduction

Ventilator-associated pneumonia (VAP) is a type of pneumonia that occurs in patients with tracheal intubation or tracheotomy after receiving mechanical ventilation for more than 48 h (1). VAP is a common complication in intensive care unit (ICU) patients, leading not only to prolonged hospital stays and increased treatment costs, but, more importantly, it can worsen the patient’s condition and even become life-threatening (2–5).

In recent years, with the increasing number of patients in intensive care units (ICUs) and the widespread use of ventilators, ventilator-associated pneumonia (VAP) has become an urgent challenge facing the global medical community. The incidence of VAP varies by region, with significantly higher rates in middle- and low-income countries compared to high-income countries (6, 7). For example, in resource-limited regions such as Southeast Asia and developing countries, the incidence rate of adult VAP ranges from 10‰ to 41.7‰ (8). In contrast, U.S. data from 2013 showed that the incidence of VAP in ICUs was only 0.8–2.2‰ (9), while the incidence in North American hospitals was 1–2.5‰ (10). However, some regions in Europe have higher incidence rates, with the EU VAP/CAP study reporting an incidence rate of 18.3‰ (11). This disparity in data highlights the close association between healthcare standards and VAP incidence rates.

The occurrence of VAP is closely associated with multiple factors, among which the knowledge, attitudes, and practices (KAP) of healthcare workers play a crucial role in VAP prevention. International studies indicate that nurses’ performance in VAP prevention is closely related to their professional knowledge level and adherence to guidelines (12). A study in Tanzania found that nurses with higher education levels demonstrated significantly better VAP prevention practices than those with lower education levels, with primary barriers including insufficient skills, staff shortages, and lack of knowledge (13). A study in Saudi Arabia further confirmed that nurses’ educational background, work experience, and gender significantly influence the implementation of VAP prevention measures (14). Australian research has pointed out that although nurses report high compliance, there is still a significant lack of awareness of evidence-based guidelines (15).

In the clinical practice of ICUs in China, the prevention and control of VAP face a unique dilemma of “discrepancy between knowledge and action.” First, the allocation of nursing resources and operational standards have a decisive impact on the effectiveness of prevention and control. A multicenter study of 32 tertiary hospitals in Hebei Province showed that the incidence of VAP was significantly associated with nurse staffing levels and operational standards (16). Second, the issue of knowledge translation barriers is prominent (17). A cross-sectional survey in Hunan Province revealed that while nurses scored high on VAP knowledge tests, their compliance rates in clinical practice were low (18). Additionally, systemic constraints are significant. A qualitative study in China identified structural factors such as physical space limitations, inadequate technical equipment, and lack of standardized training (19), which prevent nurses from implementing standardized procedures even when they possess theoretical knowledge. Finally, the sustainability of intervention measures faces challenges (20). Furthermore, existing evidence is mostly from developed regions in the east, and there is a lack of adaptive solutions for resource-constrained regions.

This study is the first to conduct a Knowledge, Attitude, and Practice (KAP) survey on VAP among ICU nurses in Gansu Province, northwest China, aiming to assess the current status of VAP prevention KAP, identify influencing factors, and propose improvement strategies based on local medical resources. Compared to existing studies, the innovative aspects of this research include: it is the first systematic investigation of VAP prevention in underdeveloped regions of western China, and it provides targeted recommendations tailored to the characteristics of medical resources in economically underdeveloped regions, thereby providing empirical evidence for the development of regionally differentiated prevention and control strategies.

## 2 Materials and methods

### 2.1 Study population

**Figure 1:**
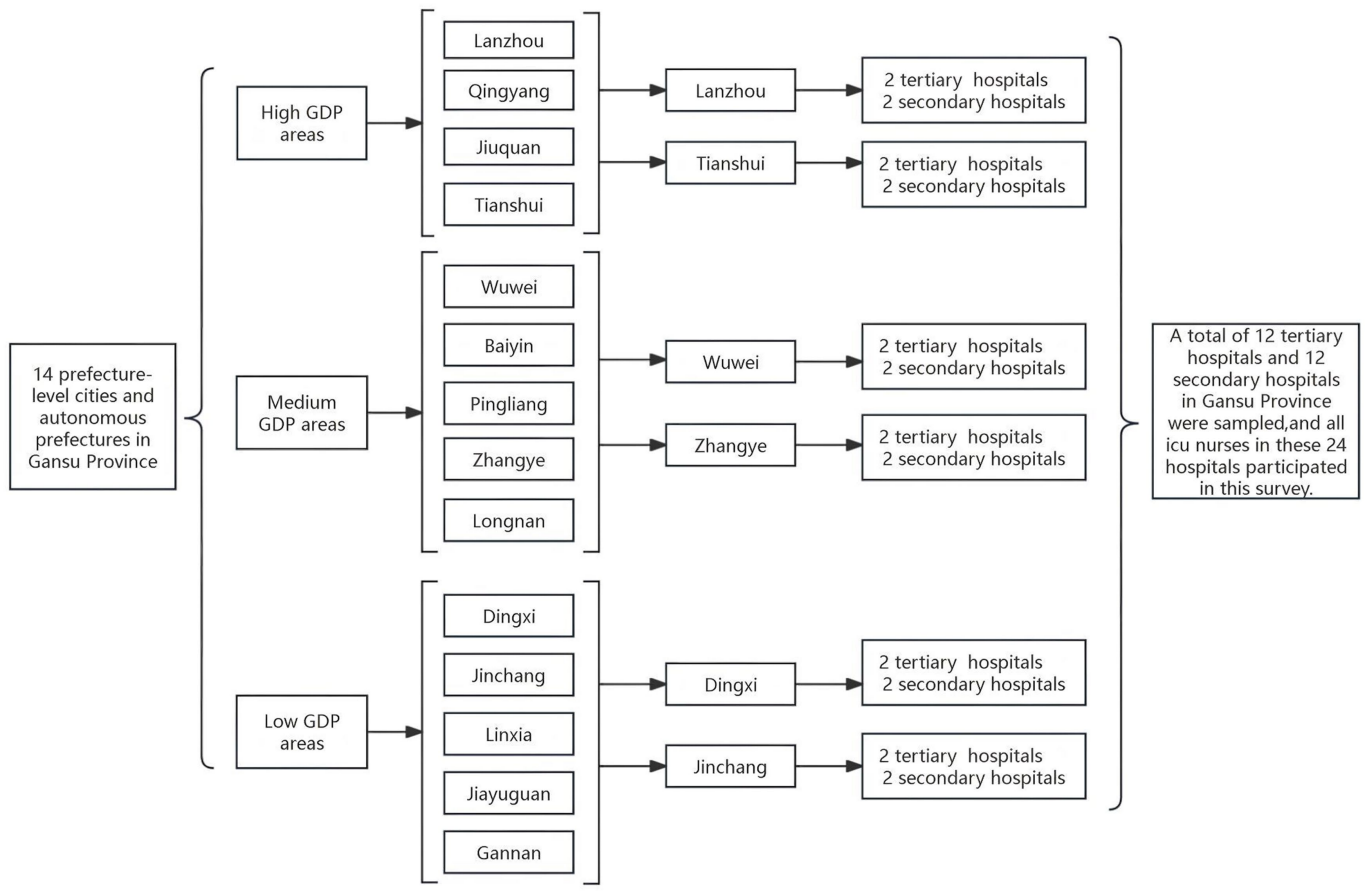
Flowchart of ICU nurse selection in Gansu province.

Using a combination of stratified random sampling and multi-stage sampling, a total of 14 prefecture-level administrative areas in Gansu Province, including 12 prefecture-level cities and 2 autonomous prefectures, were categorized into three groups based on the 2023 regional gross domestic product (GDP): high GDP, medium GDP, and low GDP. Two cities (or prefectures) were randomly selected from each group. In each of the six selected cities (or prefectures), two tertiary hospitals and two secondary hospitals were chosen, resulting in a total of 24 hospitals. A total of 600 ICU nurses from all 24 hospitals, sampled between January and December 2023, were selected as the study population (Figure 1).

### 2.2 Data collection tools

#### 2.2.1 Questionnaire design

In this study, we referred to relevant literature and guidelines on VAP prevention both domestically and internationally, particularly the Chinese Guidelines for the Diagnosis and Treatment of Hospital-Acquired Pneumonia and Ventilator-Associated Pneumonia in Adults (2018 edition), developed by the Critical Care Medicine Branch of the Chinese Medical Association, as well as the VAP prevention guidelines issued by the Centers for Disease Control and Prevention. Based on this, we extensively reviewed relevant research materials and literature from both domestic and international sources to gain an in-depth understanding of the latest advancements and practical experience in VAP prevention. At the same time, we considered the local characteristics and realities of Gansu Province, while also taking into account China’s national conditions. To ensure the scientific validity and rationality of the questionnaire, we consulted experts in the fields of ICU nursing, infection control, nursing, and statistics, incorporating their valuable insights. Through this series of research and discussions, the questionnaire for this study was ultimately developed.

#### 2.2.2 Composition of the questionnaire

The questionnaire for this study consisted of two parts: the first part was a general information survey of nurses, and the second part was the Knowledge, Attitude, and Practice (KAP) Questionnaire for ICU nurses to prevent VAP. The KAP Questionnaire was divided into three dimensions: knowledge, attitude, and practice, with a total of 44 items. The score range for the KAP Questionnaire was 22–120 points. The knowledge dimension contained 10 items, the attitude dimension had 6 items, and the practice dimension had 16 items. The knowledge dimension consisted of multiple-choice questions, with a score of 1 for a correct answer and 0 for an incorrect answer, giving a score range of 0–10 points. The attitude dimension was scored using a Likert 5-point scale, with the options “strongly agree,” “neutral,” “relatively agree,” “disagree,” and “strongly disagree” in descending order. The corresponding scores were 5, 4, 3, 2, and 1, respectively, resulting in a score range of 6–30 points. The practice dimension was also scored on a Likert 5-point scale, with the options “very compliant,” “basic compliant,” “basic noncompliant,” “noncompliant,” and “very noncompliant,” with scores of 5, 4, 3, 2, and 1, respectively. This dimension had a score range from 16 to 80 points.

#### 2.2.3 Questionnaire pre-survey

This study employed a random sampling method to select 60 ICU nurses for a preliminary survey, systematically evaluating the reliability and validity indicators of the questionnaire. The reliability analysis results showed that the Cronbach’s *α* coefficients for the belief scale and behavior scale were 0.869 and 0.808, respectively, while the Cronbach’s *α* coefficient for the total scale was 0.792. All indicators exceeded the industry-recognized standard of 0.70, confirming that the questionnaire has ideal internal consistency. Validity testing results showed that the overall content validity index (CVI) of the questionnaire reached 0.91, significantly exceeding the critical requirement of 0.80; exploratory factor analysis (EFA) revealed a KMO value of 0.950, and Bartlett’s sphericity test results were significant (*p* < 0.05), The two extracted common factors collectively explained 75.1% of the variance, with all items having factor loadings exceeding 0.5 and no cross-factor loadings observed. Based on these measurement indicators, the questionnaire developed in this study exhibits good reliability and validity characteristics, meeting measurement requirements.

### 2.3 Data collection

This study was conducted in close coordination with all participating hospitals and received approval and support from hospital management. The study employed a questionnaire distribution method, targeting ICU nurses across all participating hospitals. Prior to data collection, researchers conducted departmental briefings to provide participants with detailed explanations of the study’s objectives, methods, and precautions. Each participant was given a verbal explanation, clearly informing them of the voluntary participation principle, data anonymization procedures, and the right to withdraw at any time without reason. The questionnaire survey commenced only after obtaining verbal informed consent. No personal identification information was recorded during questionnaire completion; only basic information such as department and years of service was retained. All questionnaires used an independent coding system (questionnaire IDs were completely separated from personal information) to ensure data anonymity. After collection, the questionnaires were immediately verified by researchers on-site and centrally stored. After electronic data entry, the original paper records were promptly destroyed. This study is a low-risk observational study that does not interfere with clinical diagnostic and treatment processes. The survey content solely pertains to healthcare professionals’ work practices and does not include patient privacy or sensitive information.

### 2.4 Statistical methods

This study utilized SPSS 26.0 software for data processing and analysis, with *p* < 0.05 indicating statistical significance. First, the Kolmogorov–Smirnov test was used to assess the normality of the data, with results showing *p* < 0.05. Given the large sample size (*N* = 600) and the fact that the absolute values of skewness for all variables were less than 2, the absolute values of kurtosis were less than 7, and no extreme outliers were detected, the basic conditions for parametric tests were still met. Descriptive statistical methods were used to analyze demographic characteristics, knowledge, belief, and behavior variables, calculating frequencies, proportions, means, and standard deviations. After testing, the data met the requirements for linear relationships and homogeneity of variance, so Pearson correlation analysis was used to explore the correlations between knowledge, beliefs, and behaviors related to VAP prevention among ICU nurses. Independent samples t-tests or one-way analysis of variance were used to compare differences in scores across knowledge, belief, and behavior dimensions among nurses with different demographic characteristics (e.g., age, years of experience, educational level, etc.). Finally, multiple linear regression analysis was employed to identify the primary factors influencing ICU nurses’ knowledge, beliefs, and behaviors regarding VAP prevention.

## 3 Results

All ICU nurses in 24 hospitals agreed to participate in this study, and a total of 600 nurses completed the questionnaire. Among the participants, females accounted for the majority, 95.1%, while males accounted for only 4.8%. In terms of age distribution, 71.6% of the nurses were between 25 and 35 years old. In terms of geographical distribution, the largest number of nurses were from Lanzhou, accounting for 22.8%, while the smallest number of nurses were from Jinchang, accounting for 7.5%. In terms of hospital type, 89.8% of the nurses worked in tertiary hospitals. The most common clinical unit was general ICU, accounting for 44.6, and 76% of the nurses had more than 5 years of work experience. In terms of education, 80% of the nurses had a bachelor’s degree or higher. In addition, 87.3% of the nurses had attended at least one trainings related to VAP prevention in the past 2 years, while 80.1% of the nurses had not published any academic papers in the past 5 years. The detailed results are shown in Table 1.

**Table 1:** Demographic characteristics (*N* = 600).

Demographic item		Frequency (%)
Gender	Male	29 (4.8)
	Female	571 (95.1)
Age	<25	33 (5.5)
	25–35	430 (71.6)
	>35	137 (22.8)
Area	Lanzhou	137 (22.8)
	Tianshui	113 (18.8)
	Wuwei	120 (20.0)
	Zhangye	115 (19.2)
	Dingxi	70 (11.7)
	Jinchang	45 (7.5)
Hospital level	Grade 3A	353 (58.8)
	Grade 3B	186 (31.0)
	Grade 2A	53 (8.8)
	Grade 2B	8 (1.3)
Type of ICU	GICU	268 (44.6)
	RICU	26 (4.3)
	NICU	61 (10.1)
	CCU	37 (6.16)
	EICU	18 (3.0)
	MICU	42 (7.0)
	SICU	61 (10.1)
Working experience	<5 years	144 (24.0)
	5–10 years	223 (37.1)
	11–15 years	166 (27.6)
	16–20 years	39 (6.5)
	>20 years	28 (4.6)
Academic qualifications	Undergraduate and above	480 (80.0)
	Three-year college education and below	120 (20.0)
Professional title	Nurse	130 (21.6)
	Nurse-practitioner	295 (49.1)
	Nurse-in-charge	161 (26.8)
	Associate chief of nursing	14 (2.3)
Nurse competence level	N0	56 (9.3)
	N1	144 (24.0)
	N2	235 (39.1)
	N3	141 (23.5)
	N4	24 (4.0)
Number of VAP-related trainings	0	76 (12.6)
	≥1	524 (87.3)
Number of beds in department	<25	395 (65.8)
	25–50	143 (23.8)
	>50	62 (10.3)
Number of papers published	0	481 (80.1)
	1–2	103 (17.1)
	>2	16 (2.6)

GICU, General ICU; SICU, Surgical Intensive Care Unit; MICU, Medical Intensive Care Unit; EICU, Emergency Intensive Care Unit; RICU, Respiratory Intensive Care Unit; NICU, Neonatal Intensive Care Unit; CCU, Coronary Care Unit.

The ICU nurses’ KAP questionnaire score for preventing VAP was 113.92 ± 8.472, of which the knowledge dimension score was 7.66 ± 1.200, the attitude dimension score was 28.67 ± 3.528, and the practice dimension score was 77.59 ± 5.839. The bottom three entries of knowledge, attitude and practice dimension scores are shown in Table 2.

**Table 2:** The three items with the lowest scores in each dimension (*N* = 600).

Dimension	Items	Scores
Knowledge	K2. Recommendations for nutritional supply in mechanically ventilated patients	0.60 ± 0.489
	K6. Recommendations for humidification devices for use in mechanically ventilated patients	0.33 ± 0.472
	K8. Frequency of suction device replacement in mechanically ventilated patients	0.20 ± 0.398
Attitude	A2. I believe that caregivers should take relevant precautions to prevent ventilator-associated pneumonia	4.78 ± 0.607
	A3. I believe that taking preventive measures will have a positive impact on the patient’s recovery and treatment outcome	4.74 ± 0.712
	A6. I think caregivers should take the initiative to get up-to-date on preventing ventilator-associated pneumonia.	4.78 ± 0.618
Practice	P4. I will perform subglottic suction for mechanically ventilated patients	4.77 ± 0.559
	P14. I will use 2% chlorhexidine for oral care of mechanically ventilated patients	4.76 ± 0.586
	P16. I will perform a ‘daily wake-up call’ for sedated patients and perform a spontaneous breathing test.	4.81 ± 0.493

The results of one-way ANOVA showed (Table 3) that there was a significant relationship between knowledge level and region (*p* = 0.026). According to the LSD test, there were significant differences in VAP (ventilator-associated pneumonia) knowledge scores among nurses in Lanzhou, Tianshui, Wuwei, Zhangye, Dingxi, and Jinchang regions. Specifically, ICU nurses in Lanzhou had significantly higher VAP knowledge than nurses in the Wuwei (*p* = 0.1013) and Dingxi (*p* = 0.033) regions; and nurses in Zhangye had significantly higher VAP knowledge than nurses in the Wuwei (*p* = 0.005) and Dingxi (*p* = 0.016) regions. In addition, there were significant differences in the knowledge of VAP prevention among nurses in different types of ICUs (*p* < 0.001). According to the results of the LSD test, nurses in comprehensive ICUs had significantly higher knowledge of VAP prevention than those in RICUs (Respiratory Intensive Care Unit) (*p* < 0.001), NICUs (Neonatal Intensive Care Unit) (*p* < 0.001), CCUs (Coronary Care Unit) (*p* < 0.001), and EICUs (Emergency Intensive Care Unit) (*p* = 0.038); nurses in RICUs had significantly higher knowledge of VAP prevention than those in MICUs (Medical Intensive Care Unit) (*p* = 0.009) and SICUs (Surgical Intensive Care Unit) (*p* = 0.016); nurses in the NICU had significantly higher knowledge of VAP prophylaxis than those in the MICU (*p* < 0.001) and SICU (*p* < 0.001); and nurses in the CCU had significantly higher knowledge of VAP prophylaxis than those in the MICU (*p* = 0.003) and SICU (*p* = 0.005). Additionally, the results of the independent t-test (Table 1) showed that there was a significant difference between nurses’ mastery of VAP knowledge and the number of training sessions they had attended (*p* < 0.001). Nurses who had attended one VAP prevention-related training session in the past 2 years demonstrated significantly higher mastery of VAP knowledge than those who had attended zero training sessions. Finally, there was also a significant CHAYI1 between nurses’ knowledge acquisition and the number of beds in the department (*p* < 0.001). According to the results of the LSD test, ICU nurses with fewer than 25 beds in the unit had significantly higher knowledge of VAP prevention than nurses with 25–50 beds (*p* < 0.001) and those with more than 50 beds (*p* < 0.001).

**Table 3:** Demographic characteristics correlation with knowledge, attitude, and practice in the area of VAP prevention (*N* = 600).

Variable		Knowledge scores	Attitude scores	Practice scores	Total scores for knowledge, attitude and practice
		Mean ± SD	Mean ± SD	Mean ± SD	Mean ± SD
Gender					
	Male	7.72 ± 1.099	28.38 ± 4.144	75.03 ± 9.803	111.14 ± 13.614
	Female	7.66 ± 1.206	28.68 ± 3.497	77.72 ± 5.546	114.06 ± 8.118
*t-value*		0.295	0.452	1.464	−1.146
*p-value*		0.261	0.651	0.154	0.261
Age					
	<25	7.64 ± 1.113	28.7 ± 2.744	77.06 ± 5.385	113.39 ± 6.887
	25–35	7.70 ± 1.197	28.6 ± 3.679	77.65 ± 5.974	113.94 ± 8.662
	>35	7.55 ± 1.230	28.87 ± 3.210	77.55 ± 5.539	113.97 ± 8.258
*F-value*		0.719	0.296	0.159	0.068
*p-value*		0.488	0.744	0.853	0.935
Area					
	Lanzhou	7.79 ± 1.245	28.21 ± 3.764	75.60 ± 8.613	111.6 ± 11.981
	Tianshui	7.68 ± 1.152	29.39 ± 2.544	78.81 ± 2.735	115.88 ± 4.544
	Wuwei	7.42 ± 1.313	28.32 ± 4.323	77.84 ± 5.340	113.58 ± 8.363
	Zhangye	7.85 ± 1.157	28.48 ± 3.460	77.67 ± 5.326	114.00 ± 7.735
	Dingxi	7.41 ± 1.083	29.11 ± 3.201	78.43 ± 4.035	114.96 ± 6.104
	Jinchang	7.76 ± 1.026	28.98 ± 2.950	78.44 ± 4.630	115.18 ± 6.923
*F-value*		2.574	2.018	4.848	3.799
*p-value*		0.026	0.074	<0.001	0.002
Hospital level					
	grade 3A	7.59 ± 1.177	28.64 ± 3.911	77.90 ± 5.657	114.13 ± 8.155
	grade 3B	7.80 ± 1.168	28.85 ± 2.743	77.38 ± 5.935	114.02 ± 8.629
	grade 2A	7.55 ± 1.422	28.42 ± 3.302	76.53 ± 6.414	112.49 ± 9.703
	grade 2B	8.38 ± 1.061	27.50 ± 3.625	75.88 ± 7.338	111.75 ± 10.334
*F-value*		2.320	0.555	1.238	0.758
*p-value*		0.074	0.645	0.295	0.518
Type of ICU					
	GICU	8.07 ± 1.068	28.71 ± 3.446	77.61 ± 6.575	114.38 ± 9.251
	RICU	7.15 ± 1.047	28.85 ± 3.120	77.15 ± 5.669	113.15 ± 8.003
	NICU	7.05 ± 1.115	28.56 ± 3.095	77.26 ± 5.399	112.87 ± 7.692
	CCU	7.14 ± 1.456	28.19 ± 3.879	76.86 ± 6.579	112.19 ± 10.429
	EICU	7.50 ± 1.295	29.06 ± 2.879	78.00 ± 4.015	114.56 ± 5.586
	MICU	7.88 ± 1.087	28.50 ± 5.260	78.60 ± 3.768	114.98 ± 6.834
	SICU	7.79 ± 1.112	28.98 ± 3.640	78.13 ± 4.606	114.90 ± 7.096
*F-value*		16.022	0.284	0.504	1.066
*p-value*		<0.001	0.945	0.806	0.382
Working experience					
	<5 years	7.70 ± 1.097	28.46 ± 3.943	77.67 ± 5.739	113.83 ± 8.201
	5–10 years	7.70 ± 1.188	28.66 ± 3.632	77.26 ± 6.570	113.62 ± 9.402
	11–15 years	7.58 ± 1.266	28.75 ± 3.243	78.26 ± 3.970	114.65 ± 6.243
	16–20 years	7.62 ± 1.350	28.26 ± 3.654	79.26 ± 8.742	110.92 ± 12.938
	>20 years	7.68 ± 1.249	29.89 ± 0.416	80.26 ± 2.707	116.64 ± 3.268
*F-value*		0.269	1.126	3.171	2.348
*p-value*		0.898	0.343	0.014	0.053
Academic qualifications					
	Undergraduate and above	7.64 ± 1.197	28.64 ± 3.586	77.64 ± 5.946	113.92 ± 8.634
	Three-year college education and below	7.73 ± 1.216	28.79 ± 3.297	77.39 ± 5.410	113.91 ± 7.822
*t-value*		−0.663	−0.428	0.419	0.017
*p-value*		0.508	0.669	0.675	0.987
Professional title					
	Nurse	7.59 ± 1.105	28.68 ± 2.944	77.12 ± 6.285	113.40 ± 8.551
	Nurse-practitioner	7.64 ± 1.237	28.37 ± 4.068	77.52 ± 6.231	113.53 ± 9.150
	Nurse-in-charge	7.72 ± 1.205	29.17 ± 2.790	78.07 ± 4.485	114.96 ± 6.656
	Associate chief of nursing	8.00 ± 1.240	29.07 ± 3.474	78.00 ± 6.917	115.07 ± 11.028
*F-value*		0.673	1.894	0.674	1.278
*p-value*		0.569	0.129	0.568	0.281
Nurse competence level					
	N0	7.73 ± 1.070	29.09 ± 2.437	77.77 ± 5.444	114.50 ± 6.619
	N1	7.61 ± 1.177	28.15 ± 4.665	76.97 ± 7.642	112.73 ± 10.971
	N2	7.63 ± 1.238	28.62 ± 3.065	77.24 ± 5.795	113.49 ± 8.270
	N3	7.62 ± 1.216	28.90 ± 3.506	78.52 ± 3.881	115.05 ± 6.781
	N4	8.25 ± 1.073	29.96 ± 0.204	78.83 ± 3.130	117.04 ± 3.237
*F-value*		1.626	1.969	1.809	2.411
*p-value*		0.166	0.098	0.125	0.048
Number of VAP-related trainings					
	0	6.39 ± 1.345	27.39 ± 4.584	75.38 ± 7.908	109.17 ± 11.395
	≥1	7.84 ± 1.104	28.85 ± 3.312	77.91 ± 5.410	114.61 ± 7.735
*F-value*		−10.732	−2.674	−2.700	−4.028
*p-value*		<0.001	0.009	0.008	<0.001
Number of beds in department					
	<25	7.83 ± 1.160	28.87 ± 2.913	77.57 ± 6.268	114.27 ± 8.850
	25–50	7.38 ± 1.168	28.26 ± 4.564	77.97 ± 4.414	113.61 ± 7.113
	>50	7.23 ± 1.311	28.34 ± 4.262	76.87 ± 5.897	112.44 ± 8.822
*F-value*		12.121	1.875	0.767	1.379
*p-value*		<0.001	0.154	0.465	0.253
Number of papers published					
	0	7.65 ± 1.221	28.68 ± 3.329	77.51 ± 6.114	113.84 ± 8.752
	1–2	7.63 ± 1.085	28.60 ± 4.375	78.18 ± 3.967	114.42 ± 6.480
	>2	8.13 ± 1.258	28.81 ± 3.487	76.19 ± 7.305	113.13 ± 11.254
*F-value*		1.246	0.033	1.039	0.296
*p-value*		0.288	0.967	0.354	0.764

GICU, General ICU; SICU, Surgical Intensive Care Unit; MICU, Medical Intensive Care Unit; EICU, Emergency Intensive Care Unit; RICU, Respiratory Intensive Care Unit; NICU, Neonatal Intensive Care Unit; CCU, Coronary Care Unit.

According to the results of the independent t-test (Table 1), there was a statistically significant difference in the beliefs of nurses with different training frequencies (*p* = 0.009). ICU nurses who had participated in VAP-related training more than once in the past 2 years had significantly stronger beliefs in VAP prevention than nurses who had participated zero times.

According to one-way ANOVA (Table 3), there was a significant difference in practice scores of VAP prevention among ICU nurses in different regions (*p* < 0.001). The practice scores of ICU nurses in VAP prevention were significantly higher in Lanzhou than in other regions. There was a significant difference in the practice scores of ICU nurses with different years of experience (*p* = 0.014), and the practice scores of nurses with 16–20 years of experience were all significantly lower than those of nurses with <5 years of experience (*p* = 0.013), 5–10 years of experience (*p* = 0.029), 11–15 (*p* = 0.002) years of experience, and >20 years of experience (*p* = 0.005).

The results of the independent samples t-test (Table 1) showed that the difference between the number of training sessions and the practice scores was also statistically significant (*p* = 0.008). ICU nurses who received one or more training sessions had significantly higher practice scores than nurses who did not receive any training.

One-way ANOVA showed (Table 3) that there was a significant difference in the total score of the questionnaire of knowing and believing in different regions (*p* = 0.002), with ICU nurses in Lanzhou having a significantly higher total score of the questionnaire of knowing and believing in ICU nurses than those in other regions, and the questionnaire of Tianshui having a significantly higher total score than those in Wuwei (*p* = 0.036). The differences between the questionnaires of nurses with different levels of competence were also statistically significant, with nurses with competence levels N3 (*p* = 0.021) and N4 (*p* = 0.021) having significantly higher total KAP questionnaire scores than nurses with competence level N1. The results of the independent t-test (Table 1) showed that there were also significant differences in the total scores of the knowledge, belief, and behavior questionnaire between nurses with different training frequencies (*p* < 0.001). Nurses who had received training one or more times had higher total scores than nurses who had not participated in training.

The results of the correlation between knowledge, attitude and practices related to VAP among ICU nurses are shown in Table 4. There was a significant positive correlation between knowledge and attitude (*r* = 0.100, *p* < 0.015). By increasing the knowledge scores, the attitude scores were also increased. Also, there was a significant positive correlation between knowledge and practice (*r* = 0.104, *p* < 0.011), with higher knowledge scores resulting in higher practice scores. There was also a significant positive correlation between attitude and practices (*r* = 0.522, *p* < 0.001), with higher attitude scores leading to higher practice scores.

**Table 4:** Correlation of knowledge, attitude, and practice related to prevention VAP of nurses in ICU.

Variable	Scores of knowledge	Scores of attitude	Scores of practice
Scores of knowledge			
Pearson’s correlation	1	0.100*	0.104*
Sig.		0.015	0.011
*N*	600	600	600
Scores of attitude			
Pearson’s correlation	0.100*	1	0.522*
Sig.	0.015		<0.001
*N*	600	600	600
Scores of practice			
Pearson’s correlation	0.104*	0.522*	1
Sig.	0.011	<0.001	
*N*	600	600	600

*p < 0.05.

The results showed that the number of trainings and region were the influencing factors of ICU nurses’ knowledge and practice in this study. The number of trainings was also an influential factor in the total score of nurses’ attitude and KAP questionnaire (Table 5).

**Table 5:** Multiple linear regression analysis of ICU nurses’ total scores on nursing knowledge, attitude, practices, and knowledge, attitude and practices regarding VAP prevention (*N* = 600).

Independent variables	*B*	*SE*	*β*	*t-value*	*p-value*
Scores of knowledge					
Constant	8.015	0.203		39.432	<0.001
Type of ICU	−0.045	0.024	−0.077	−1.886	0.060
Number of VAP-related trainings	0.166	0.061	0.109	2.701	0.007
Number of beds in department	−0.047	0.031	−0.061	−1.503	0.133
Area	−0.306	0.072	−0.172	−4.224	<0.001
Scores of attitude					
Constant	27.448	0.410		66.964	<0.001
Number of VAP-related trainings	0.576	0.181	0.129	3.176	0.002
Scores of practice					
Constant	74.476	0.929		80.196	<0.001
Working experience	0.067	0.225	0.012	0.297	0.767
Number of VAP-related trainings	0.852	0.302	0.115	2.818	0.005
Area	0.385	0.153	0.103	2.516	0.012
Total scores for knowledge, attitude and practice					
Constant	107.738	1.452		74.219	<0.001
Nurse competence level	0.587	0.344	0.069	1.704	0.089
Number of VAP-related trainings	1.525	0.44	0.142	3.469	0.001
Area	0.417	0.221	0.077	1.886	0.06

## 4 Discussion

According to the results of this study, ICU nurses demonstrated relatively insufficient knowledge regarding the prevention of VAP. Specifically, the nurses’ knowledge dimension score (7.66 ± 1.200) was low, indicating that there is still significant room for improvement in their understanding of VAP prevention-related knowledge. These findings are consistent with those of other domestic and international studies (21, 22). However, some studies have found that ICU nurses have a good understanding of knowledge related to VAP prevention (18). In this study, ICU nurses from different regions demonstrated varying levels of mastery of VAP prevention knowledge, particularly those from Lanzhou and Zhangye, whose knowledge scores were significantly higher than those from other regions. This may be attributed to differences in medical resource allocation, hospital management systems, and training opportunities available to nurses in these regions. In contrast, some regions may invest less in VAP prevention training, leading to deficiencies in nurses’ learning and mastery of related knowledge. In this study, nurses who had received VAP training demonstrated significantly better mastery of VAP knowledge than those who had not, a finding consistent with the results of several other scholars’ studies (18, 21, 23). Jordanian scholars conducted an observational study of 428 nurses working in intensive care units (24), finding that after receiving relevant training and education, nurses’ understanding of VAP prevention knowledge improved. Secondly, the type of ICU is also an important factor influencing nurses’ knowledge of VAP prevention. Nurses in general ICUs demonstrated significantly higher levels of VAP prevention knowledge compared to nurses in other types of ICUs. This is closely related to the clinical characteristics of ICUs, as general ICUs typically admit more complex cases, presenting nurses with greater clinical challenges. Additionally, numerous domestic and international studies have found that factors such as nurses’ educational background (21), years of ICU work experience, hospital level, and age (18) also influence ICU nurses’ VAP knowledge levels. However, these factors were not significant in this study.

According to the results of this study, ICU nurses generally have a positive attitude toward VAP prevention (mean attitude score: 28.67 ± 3.528), which is consistent with the findings of Li Suwen et al. in their study of 291 ICU nurses in Ganzhou City (17). Notably, nurses who had undergone specialized VAP training scored significantly higher than those who had not (*p* < 0.05). This finding holds important practical implications for healthcare settings with limited resources.

The results of this study indicate that ICU nurses demonstrate generally adequate performance in VAP prevention practices (mean score: 77.59 ± 5.839). This finding differs from the conclusions of a study conducted in Ethiopia (25), and such discrepancies may stem from systemic factors such as the allocation of medical resources and the sophistication of training systems. Notably, nurses who had received VAP training in the past 2 years demonstrated significantly better practice performance than those who had not received training. This result aligns with findings from a study at a tertiary care center in western India (26), further confirming the critical role of training interventions in improving clinical practice. The study also found significant differences in practice levels among ICU nurses across different regions, which may be related to variations in healthcare resource allocation, the completeness of training systems, and standardized quality control frameworks in these regions.

Based on the research findings, it is recommended to establish a tiered training system in resource-limited ICU settings. For junior nurses, foundational training should be conducted using the “1 + X” model, while senior nurses should receive advanced training such as updates on evidence-based guidelines. These efforts should be complemented by low-cost interventions such as daily knowledge point push notifications, peer micro-training, and visual checklists. At the management level, VAP prevention should be incorporated into performance evaluations, with priority given to ensuring adequate basic equipment configuration, and regional demonstration centers should be established to promote resource sharing. Future research should focus on comparing the effectiveness of different training models, analyzing practical barriers, and evaluating the long-term effects of intervention measures to continuously optimize VAP prevention strategies.

## 5 Conclusion

Most ICU nurses in Gansu Province performed well in terms of their attitude toward and practice of VAP prevention and were able to implement preventive and control measures effectively. However, there were deficiencies in their knowledge acquisition related to VAP prevention. Factors such as regional differences, ICU type, and the number of training sessions significantly impacted nurses’ VAP prevention and control abilities. In particular, the frequency of training sessions and regional resource allocation played a key role in nurses’ professional competence. A significant positive correlation was found between knowledge, attitude, and practice of VAP prevention, suggesting that enhanced knowledge training could improve nurses’ attitudes and practices. Therefore, hospitals should focus on providing regular training, especially in areas with limited knowledge and fewer training opportunities, to improve nurses’ comprehensive prevention and control competence.

## Data Availability

The original contributions presented in the study are included in the article/Supplementary material, further inquiries can be directed to the corresponding authors.
