# Supplementary material for: Knowledge, attitude, and practice of nurses in ICU on preventing ventilator-associated pneumonia: a cross-sectional study in Gansu Province, China
Source: Front Med (Lausanne). 2025 Jul 9;12:1591582. doi: 10.3389/fmed.2025.1591582 (PMC12283992; doi:10.3389/fmed.2025.1591582)
Supplement: Supplementary file 1 [file Table_1.DOCX]

**Survey Questionnaire on the Current Status of Knowledge, Beliefs, and Practices Regarding the Prevention of Ventilator-Associated Pneumonia in Mechanically Ventilated Patients Among ICU Nurses in Gansu Province**

Dear Nurse Teacher:

Hello! This questionnaire aims to understand your views and practical experiences regarding the prevention of ventilator-associated pneumonia (VAP) in mechanically ventilated patients. The findings will provide reference for future research on preventive strategies for VAP in mechanically ventilated patients and further enhance your understanding of VAP-related knowledge. Please complete each section of the questionnaire honestly based on your actual experience and opinions. There are no right or wrong answers. The survey data will be used solely for academic research purposes, and your personal information will be kept strictly confidential.

**Instructions:** Please mark the corresponding number with a “√” and fill in the blank spaces with the appropriate information.

**I. General Information**

1. Your gender

① Male ② Female

2. Your age

① Under 25 ② 25–35 years old ③ Over 35 years old

3. The grade of your hospital

① Grade III Level A ② Grade III Level B ③ Grade II Level A ④ Grade II Level B ⑤ Other

4. The type of your ICU

①General ICU ②Surgical Intensive Care Unit (SICU) ③Medical Intensive Care Unit (MICU) ④Emergency Intensive Care Unit (EICU) ⑤Respiratory Intensive Care Unit (RICU) ⑥Neonatal Intensive Care Unit (NICU) ⑦Coronary Care Unit (CCU) ⑧Other

5. Your years of work experience

①＜5 years ②5–10 years ③11–15 years ④16–20 years ⑤＞20 years

6. Your educational background

①Vocational school ②Junior college ③Bachelor's degree ④Master's degree or above

7. Your professional title

①Nurse ②Registered Nurse ③Senior Registered Nurse ④Deputy Chief Nurse ⑤Chief Nurse

8. Your nursing level

①N0 ②N1 ③N2 ④N3 ⑤N4

9. Number of times you have participated in training related to the prevention of ventilator-associated pneumonia in the past two years:

10. Number of beds in your department:

11. Number of papers you have published in the past five years:

**II. Knowledge, Beliefs, and Practices Questionnaire**

**(1) Knowledge (Single Choice)**

1. Recommended method for endotracheal intubation

① Nasal endotracheal intubation ② Oral endotracheal intubation ③ Don’t know

2. Nutritional supply method for mechanically ventilated patients

① Enteral nutrition ② Parenteral nutrition ③ Don’t know

3. Recommended position for mechanically ventilated patients

① Prone position ② Head of bed elevated 30°–45° ③ Don’t know

4. The cuff pressure of the endotracheal tube should not be less than

①25–30 cmH₂O ②30–35 cmH₂O ③I don't know

5. How should condensate from the condenser tube be disposed of?

①Dispose of as medical waste ②Pour into the sink ③I don't know

6. Recommended humidification device for mechanically ventilated patients

①Heated humidification ②Heat exchanger ③I don't know

7. Recommended suctioning device for mechanically ventilated patients

① Closed suctioning device ② Open suctioning device ③ Unknown

8. Replacement frequency of suctioning devices for mechanically ventilated patients

① Replace daily ② No need to replace daily if no damage or contamination ③ Unknown

9. Ventilator tubing for mechanically ventilated patients

① One person, one use, one disinfection ② Can be used by multiple people ③ Unknown

10. Can subglottic secretion suctioning prevent aspiration and reduce the risk of VAP?

① Can prevent ② Cannot prevent ③ Don't know

**(II) Attitude**

1. I believe nursing measures are very important for preventing ventilator-associated pneumonia

① Strongly agree ② Somewhat agree ③ Neutral ④ Disagree ⑤ Strongly disagree

2. I believe nursing staff should take relevant preventive measures to prevent the occurrence of ventilator-associated pneumonia

① Strongly agree ② Somewhat agree ③ Neutral ④ Disagree ⑤ Strongly disagree

3. I believe that implementing preventive measures will have a positive impact on patients' recovery and treatment outcomes

① Strongly agree ② Somewhat agree ③ Neutral ④ Disagree ⑤ Strongly disagree

4. I will actively provide nursing care for mechanically ventilated patients to prevent ventilator-associated pneumonia

① Strongly agree ② Somewhat agree ③ Neutral ④ Disagree ⑤ Strongly disagree

5. I believe that nursing staff should actively participate in professional training on preventing ventilator-associated pneumonia

① Strongly agree ② Somewhat agree ③ Neutral ④ Disagree ⑤ Strongly disagree

6. I believe that nursing staff should proactively seek out the latest knowledge on preventing ventilator-associated pneumonia

① Strongly agree ② Somewhat agree ③ Neutral ④ Disagree ⑤ Strongly disagree

**(III)Practice**

1. I wash my hands or change gloves before and after contact with different patients

①Very compliant ②Basically compliant ③Basically non-compliant ④Non-compliant ⑤Very non-compliant

2. I use alcohol-based hand sanitizer or soap and water when washing my hands

①Very compliant ②Basically compliant ③Basically non-compliant ④Non-compliant ⑤Very non-compliant

3. I elevate the head of the bed by 30°–45° for mechanically ventilated patients without contraindications

① Strongly agree ② Basically agree ③ Basically disagree ④ Disagree ⑤ Strongly disagree

4. I perform subglottic suctioning for mechanically ventilated patients

① Strongly agree ② Basically agree ③ Basically disagree ④ Disagree ⑤ Strongly disagree

5. I promptly suction secretions for mechanically ventilated patients

①Very compliant ②Basically compliant ③Basically non-compliant ④Non-compliant ⑤Very non-compliant

6. I would regularly turn, pat the back, and perform vibration to help patients on mechanical ventilation without contraindications clear their secretions

①Very compliant ②Basically compliant ③Basically non-compliant ④Non-compliant ⑤Very non-compliant

7. I would strictly adhere to aseptic technique when performing airway care for patients with tracheostomy

①Very compliant ②Basically compliant ③Basically non-compliant ④Non-compliant ⑤Very non-compliant

8. I will provide enteral nutrition for mechanically ventilated patients

①Very compliant ②Basically compliant ③Basically non-compliant ④Non-compliant ⑤Very non-compliant

9. I will replace the ventilator tubing for mechanically ventilated patients once a week

①Very compliant ②Basically compliant ③Basically non-compliant ④Non-compliant ⑤Very non-compliant

10. When the ventilator tubing of a mechanically ventilated patient has visible stains or malfunctions, I will replace it promptly.

①Very compliant ②Basically compliant ③Basically non-compliant ④Non-compliant ⑤Very non-compliant

11. I will prevent condensation from the ventilator tubing from entering the lower respiratory tract or humidifier.

①Very compliant ②Basically compliant ③Basically non-compliant ④Non-compliant ⑤Very non-compliant

12. I will assess the oral hygiene status of mechanically ventilated patients daily.

①Very compliant ②Basically compliant ③Basically non-compliant ④Non-compliant ⑤Very non-compliant

13. I will perform oral care for mechanically ventilated patients every 4-6 hours.

①Very compliant ②Basically compliant ③Basically non-compliant ④Non-compliant ⑤Very non-compliant

14. I will use 2% chlorhexidine to perform oral care for mechanically ventilated patients.

①Very compliant ②Basically compliant ③Basically non-compliant ④Non-compliant ⑤Very non-compliant

15. I will perform early rehabilitation exercises for mechanically ventilated patients

①Very compliant ②Basically compliant ③Basically non-compliant ④Non-compliant ⑤Very non-compliant

16. I will perform “daily awakening” for sedated patients and conduct spontaneous breathing tests

①Very compliant ②Basically compliant ③Basically non-compliant ④Non-compliant ⑤Very non-compliant
